# Investigation of de novo cholesterol synthetic capacity in the gonads of goldfish (Carassius auratus) exposed to the phytosterol beta-sitosterol

**DOI:** 10.1186/1477-7827-4-60

**Published:** 2006-11-21

**Authors:** Rainie L Sharpe, Melissa Drolet, Deborah L MacLatchy

**Affiliations:** 1 Canadian Rivers Institute and Department of Biology, University of New Brunswick, PO Box 5050, 100 Tucker Park Road, Saint John, NB, E2L 4L5, Canada; 2 Department of Physical Sciences University of New Brunswick, PO Box 5050, 100 Tucker Park Road, Saint John, NB, E2L 4L5, Canada

## Abstract

Total and intra-mitochondrial gonadal cholesterol concentrations are decreased in fish exposed to the phytoestrogen beta-sitosterol (beta-sit). The present study examined the potential for beta-sit to disrupt de novo cholesterol synthesis in the gonads of goldfish exposed to 200 microgram/g beta-sit and 10 microgram/g 17beta-estradiol (E2; estrogenic control) by intra-peritoneal Silastic^® ^implants for 21 days. The de novo cholesterol synthetic capacity was estimated by incubating gonadal tissue with 14C-acetate for a period of 18 hours, followed by chloroform/methanol lipid extraction and thin layer chromatography (TLC) lipid separation. Lipid classes were confirmed using infrared spectroscopy. Plasma testosterone (T) and total cholesterol concentration were measured and gonadosomatic index (GSI) was calculated. Plasma T was significantly reduced in male beta-sit-treated fish compared to control and E2-treated fish (p < 0.001). 14C-Acetate incorporation into cholesterol and cholesterol esters was not significantly different among treatment groups for male and female fish, however, 14C-enrichment was higher than expected in both triglycerides (TG) and free fatty acids (FFA). FFA incorporation was significantly higher in male control fish than either beta-sit or E2 treatments (p = 0.005). Plasma cholesterol concentration was significantly increased in the male beta-sit treatment group compared to controls (p = 0.027). These results indicate gonadal de novo cholesterol biosynthetic capacity is not disrupted by beta-sit or E2 treatment in early recrudescing male or female goldfish, while plasma cholesterol and steroid concentrations are sensitive to beta-sit exposure.

## Background

Cholesterol is the precursor to all steroid hormones, such as estrogens, androgens, and corticosteroids [1]. In fish, as in other vertebrate species, cholesterol is obtained by dietary intake, release from intracellular stores or by *de novo *synthesis [2]. Cholesterol absorbed at the intestines is esterified with free fatty acids to form hydrophobic cholesterol esters (CEs), which are transported in the plasma in association with lipoproteins to sites of metabolism or storage [3,4]. At the tissues, receptor-mediated lipoprotein endocytosis delivers cholesterol to the intracellular environment for immediate use or re-esterification for intracellular storage [5]. While exogenous cholesterol is obtained in this way by most steroidogenic tissues (such as the adrenal and ovary), some tissues (such as the testis) differentially utilize *de novo *synthesized cholesterol as a substrate for steroid biosynthesis [2,6]. The definitive mechanisms of cholesterol metabolism in fish are believed to be highly similar to mammals, although comprehensive information on lipid dynamics in teleost species is limited. Cholesterol biosynthesis is believed to proceed by the same pathway in fish as in higher vertebrates [7]. A number of reviews on animal [6,8,9] and fish [10] lipoprotein dynamics indicate structure is similar, although TG content is elevated at the expense of cholesterol esters in teleost lipoproteins. Fish plasma is considered hypercholesterolemic relative to higher vertebrates, with 2–6× higher circulating cholesterol concentrations a normal physiological condition in fish species [11]. The traditional detrimental affects associated with high plasma cholesterol such as coronary lesions and plaque formation are notably absent in fish species, presenting an interesting and stark contrast to the human condition.

**Figure 1:**
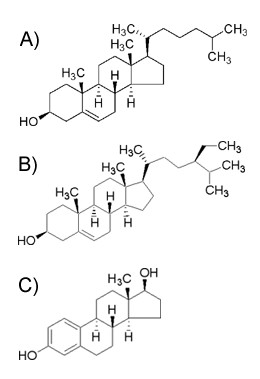
Chemical structures of (A) cholesterol (B) β-sitosterol (β-sit), and (C) 17β-estradiol (E_2_).

*De novo *cholesterol synthesis begins with the production of acetyl CoA via acetate or citrate. Acetyl CoA is subsequently transformed to 3-hydroxy-3-methylglutaryl-CoA (HMG CoA), mevalonate, squalene, lanosterol and ultimately cholesterol via a number of enzymatic transformations (for a detailed summary see [12]). The newly synthesized cholesterol (Figure 1A) can then enter tissue-specific biosynthetic pathways such as steroidogenesis, or be incorporated into plasma membranes or esterified for intracellular storage. The relative contribution of *de novo *cholesterol synthesis to the total cholesterol pool in endocrine organs such as the gonads is generally not known. Ovarian tissue in mammals has been shown to utilize *de novo *TG and cholesterol synthesis during the pre-ovulatory phase of gonadal development, doing so preferentially over lipoprotein uptake [2,13]. Testis cholesterol is preferentially sourced from *de novo *synthesis, while the adrenal and ovary appear to revert to endogenous synthesis only if circulating concentrations are limiting [2]. Preferences of lipoprotein-derived or *de novo *synthesized cholesterol by fish endocrine tissues are presumably similar to mammalian tissues, however, direct studies on fish species are lacking.

The phytosterol β-sitosterol (β-sit) very closely resembles cholesterol, differing only by an ethyl group on carbon 24 (Figure 1B). β-sit disrupts normal endocrine system function in fish by decreasing steroidogenic biosynthetic capacity [14] and disrupting plasma cholesterol concentrations and mitochondrial translocation to the steroidogenic pathway [15,16]. β-Sit has estrogenic properties as evidenced by the induction of the normally quiescent vitellogenin gene in male fish [17]. The capacity for plant sterols to affect *de novo *cholesterol synthesis is known to occur in the human disorder sitosterolemia, a condition where abnormally high concentrations of plant sterols accumulate in the plasma and tissues. Sitosterolemic patients experience impaired whole body *de novo *cholesterol synthesis by down-regulation of key synthetic enzymes [18,19], and this sensitivity suggests *de novo *cholesterol synthesis in other species may also be impaired by phytosterol exposure. Previous studies have identified changes in cholesterol availability following phytosterol exposure [12], however, a lack of information on preferred substrate (exogenous or *de novo *synthesized cholesterol) in the gonads impedes further studies to determine mechanisms of endocrine disruption.

The present study examined endogenous cholesterol synthesis in the gonads of male and female goldfish exposed to β-sit and 17β-estradiol (an estrogenic control; Figure 1C) to determine if *de novo *cholesterol synthetic capacity was impaired relative to non-exposed fish. Further, the relative contribution of *de novo *cholesterol synthesis to the reproductive steroidogenic pathway is unknown in fish; therefore, this study also aimed to assess the contribution of *de novo *synthesis to the total cholesterol substrate pool.

## Methods

All chemicals were purchased from Sigma-Aldrich (Oakville, ON, Canada) unless otherwise specified.

### Fish

Goldfish (*Carassius auratus*) were purchased from Aleong International (Mississauga, ON, Canada) and acclimated to lab conditions in 66-L flow-through tanks (10–16°C de-chlorinated City of Saint John water). During holding, fish were held on a 12:12 light:dark photoperiod and fed commercial trout pellets *ad libitum *(Corey Feed Mills, Fredericton, NB, Canada) every other day. Fish were transferred to experimental tanks two weeks prior to the start of the experiment.

### Implants

Fish were exposed to 200 μg/g β-sit (97% pure synthetic β-sitosterol, catalogue # S1270) or 10 μg/g 17β-estradiol (E_2_; catalogue # E8875) via Silastic^® ^implants. This mode of *in vivo *dose delivery has been established as an effective exposure route for goldfish [15,16] and implants have been shown to continuously release consistent doses over time [20,21].

### Exposures

Fish were allocated among the tanks such that there were 12 fish per tank during the exposure, with a random sex ratio (tubercles were not visible for sexing at the time of implanting). Fish were anaesthetized in 0.05% TMS (tricaine methane sulfonate; Syndel International, Vancouver, BC, Canada). Fish weights and lengths were recorded followed by intra-peritoneal implanting of the Silastic^® ^pellets containing either 0 μg/g (control), 200 μg/g β-sit or 10 μg/g E_2_.

During the exposure, fish were held at 15–16°C and 14:10 light:dark photoperiod. The fish were fed 1.5% body weight daily during the exposure.

At the time of sampling, fish had been implanted for 21 days. Fish were bled by caudal puncture and plasma was separated by centrifugation and stored at -20°C until steroids were extracted and cholesterol was measured. Weights (0.1 g) and lengths (mm) were recorded and the gonads were removed, weighed (0.01 g) and immediately used in the *de novo *incubation. Following the incubation, gonads were frozen at -80°C until the cholesterol assay was performed. Gonadosomatic indices (GSI) were calculated as per the equation:

GSI (%) = [gonad weight (g)/body weight (g)] × 100.

### Radioimmunoassay

Plasma hormones were extracted and testosterone (T) concentrations were measured by radioimmunoassay (RIA) [22]. A 45-minute incubation was performed at 4°C after addition of 200 μL of charcoal solution and prior to the 12-minute centrifugation at 1900 *g*. This extra step was added to the protocol to stabilize and standardize counts throughout the assay. Radio-labelled [1,2,6,7-^3^H]-testosterone was purchased from Amersham-Pharmacia (Baie d'Urfé, QC, Canada). Antibodies to T were purchased from Medicorp (Montreal, QC, Canada) and cross-reactivity is reported as 35% with dehydroepitestosterone and <0.1% with other major steroids [23]. Both intra- and inter-assay variability were within acceptable limits (< 10%).

### De novo incubation

Approximately 25 mg of gonadal tissue were cut into two pieces and placed in wells on a 24-well cell cluster plate (performed in duplicate for each gonad; Fisher Scientific, Nepean, ON, Canada). Each well received 1 mL of incubation solution which consisted of Medium 199 (M199; Sigma catalogue #M2520) containing 5 μCi of acetic acid-UL-^14^C (catalogue #314641). The plates were incubated for 18 hours at 18°C after which the incubation solution was removed and counted for total radioactivity. The samples were washed with 1.5 mL of wash solution (M199 and 2 mM unlabelled sodium acetate) which was removed and counted for total radioactivity. The tissue samples were stored at -80°C until the cholesterol assay was performed.

### Cholesterol assay

Cholesterol was extracted from gonadal tissue using a modified chloroform/methanol extraction method [24]. In brief, samples were homogenized in liquid N_2 _using a mortar and pestle. Lipids were subsequently extracted by adding 3.5 mL chloroform, 4.5 mL methanol and 2 × 10^4 ^dpm 1α, 2α [N]-^3^H-cholesterol (for recovery estimation; #C8794) to each sample. The tubes were mixed and left to settle before adding an additional 2 mL of chloroform. The tubes were mixed and left to settle before adding 3 mL of 2 M KCl with 5 mM EDTA. Once settled, the bottom phase was transferred to a new test tube and washed twice with a 1:2 mix of methanol: 0.9% NaCl. The chloroform was evaporated under N_2 _gas and the samples were re-suspended in 40 μl of chloroform for use in thin-layer chromatography (TLC).

Samples were spotted (10 μl) on Whatman LK5DK linear plates (Fisher Scientific), with a chloroform-only and cholesterol standard control run concurrently on each plate. The plates were put through two phases of development in separate chambers in a method modified from [25]. Phase 1 consisted of chloroform: methanol: acetic acid (98:2:1) and was developed up to 17 cm. Phase 2 consisted of hexane: ethyl ether: acetic acid (96:4:0.2) and was developed to the top of the plate. Plates were left to dry and areas corresponding to lipids were identified by exposure to iodine vapour and marked. After 2–12 h (when iodine disappeared) the spots corresponding to lipids were scraped into scintillation vials and counted for ^3^H- and ^14^C-radioactivity.

### Lipid classification

The spot corresponding to cholesterol (chol) was first identified by co-migration with the cholesterol standard, and subsequently confirmed by infrared spectroscopy (IR). Lipids were classified using IR; spots corresponding to free fatty acid (FFA), triglyceride (TG), and cholesterol ester (CE) were examined using IR and confirmed by comparison to the separation of simple lipids by similar solvent systems [26].

### Plasma cholesterol

Total plasma cholesterol concentration was measured using a commercially available spectrophotometric assay (CIMA Scientific, De Soto, TX, USA). A 10 μl volume of plasma was added to 1 mL of colour reagent and incubated at 37°C for 10 min. The absorbance was read at 515 nm and the concentration of the unknown samples was calculated in comparison with a calibration standard.

### Statistical analysis

Treatment differences were tested using a nested analysis of variance (ANOVA; done manually using Microsoft Office Excel 2003) with tank as the nesting factor. If treatment differences were present (p < 0.05) and the data were parametric, Holm-Sidak tests were performed using Sigmastat 3.0 (SPSS Corp, Chicago, IL) to identify the different treatment groups. If the data were non-normal or had unequal variances, non-parametric Dunn's tests were performed. Systat 10.2 (SPSS Inc, Chicago, IL) was used to perform analysis of covariance (ANCOVA) on gonad and body weight data to determine if there were differences in gonad size among treatment groups.

## Results

### GSI

Male and female fish had developing gonads at the early to mid-recrudescence stage, as evidenced by the state of the gonads at the time of sampling. There were no differences in gonad weight relative to body weight as compared to controls in male and female fish. Male GSI values, mean ± SEM (N), were 1.10 ± 0.2 (9), 0.7 ± 0.3 (11) and 1.78 ± 0.3 (11) for control, β-sit and E_2 _treatment groups, respectively. Male β-sit-treated fish had significantly smaller gonads than the E_2 _treatment group (p = 0.032). The GSI values for female control, β-sit and E_2 _treated-fish were 2.80 ± 0.8 (15), 1.9 ± 0.5 (13) and 2.46 ± 0.4 (13), respectively.

### Plasma testosterone

**Figure 2:**
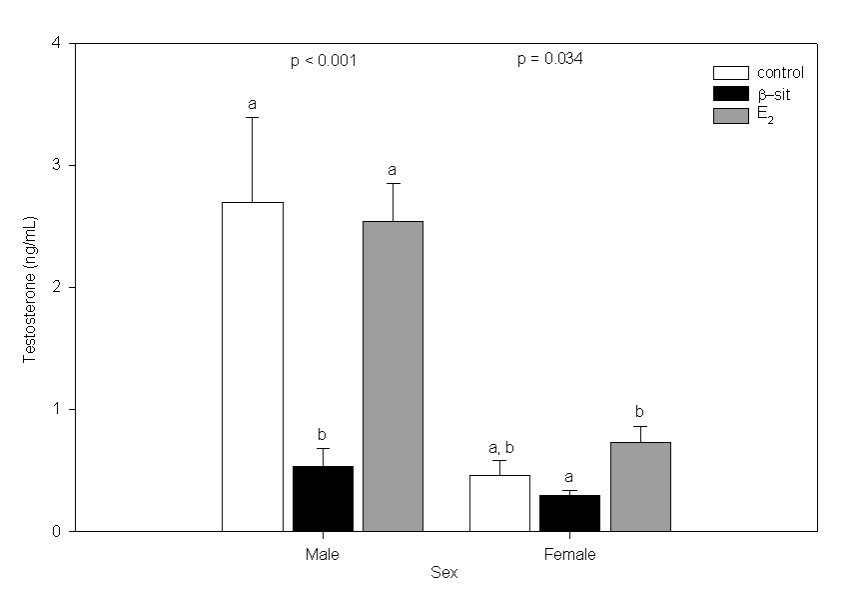
Plasma testosterone concentration (ng/mL) in male and female goldfish exposed to control, β-sit (200 μg/g) and E_2 _(10 μg/g) via Silastic^® ^implants for 21 days. Bars represent means ± SE. Different letters indicate treatments that are significantly different (p < 0.05).

β-Sit significantly reduced plasma T concentrations in male fish relative to controls (p < 0.001; Figure 2), while plasma T concentrations of β-sit-exposed females were not different from those of control fish. The β-sit treated females had significantly lower plasma T than the E_2 _treatment group (p = 0.034).

### De novo cholesterol assay

**Figure 3:**
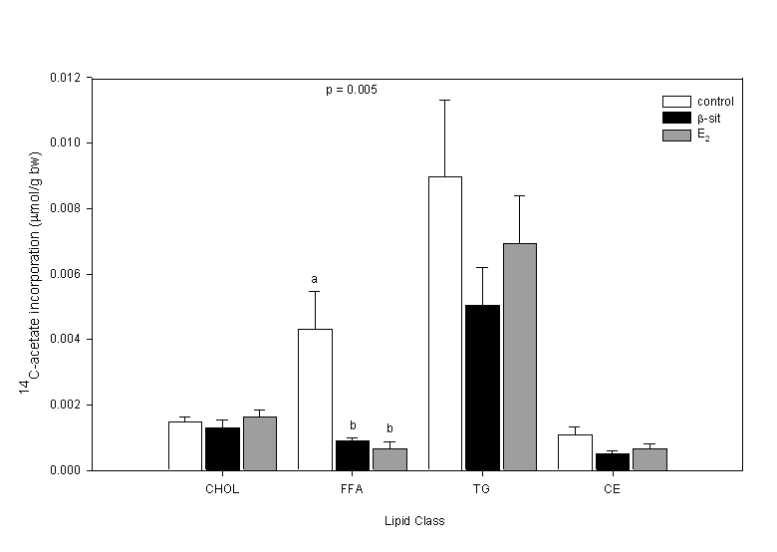
Gonad lipid ^14^C-acetate incorporation (μmol/g bw) toward cholesterol (chol), free fatty acid (FFA), triglyceride (TG) and cholesterol ester (CE) in male goldfish exposed to control, β-sit (200 μg/g) and E_2 _(10 μg/g) via Silastic^® ^implants for 21 days. Values are presented as means ± SE. Different letters indicate treatments that are significantly different (p < 0.05).

**Figure 4:**
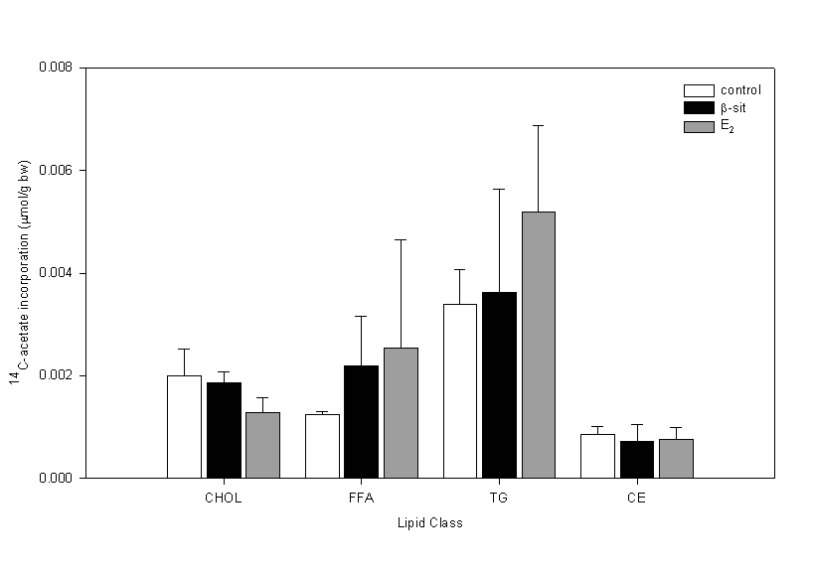
Gonad lipid ^14^C-acetate incorporation (μmol/g bw) towards cholesterol (chol), free fatty acid (FFA), triglyceride (TG) and cholesterol ester (CE) in female goldfish exposed to control, β-sit (200 μg/g) and E_2 _(10 μg/g) via Silastic^® ^implants for 21 days. Values are presented as means ± SE. There are no treatment differences (p > 0.05).

The cholesterol extraction protocol recovered 90.7 ± 0.6% of the ^3^H-cholesterol, indicating a high extraction efficiency. There were no differences in ^14^C-acetate incorporation into cholesterol or CE in male (Figure 3) or female (Figure 4) fish. There was significantly higher ^14^C-acetate incorporation into FFAs in male control fish than in either β-sit or E_2 _treatment groups. There were no treatment differences for either sex in cholesterol: CE or cholesterol: TG ratio (data not shown).

### Plasma cholesterol

**Figure 5:**
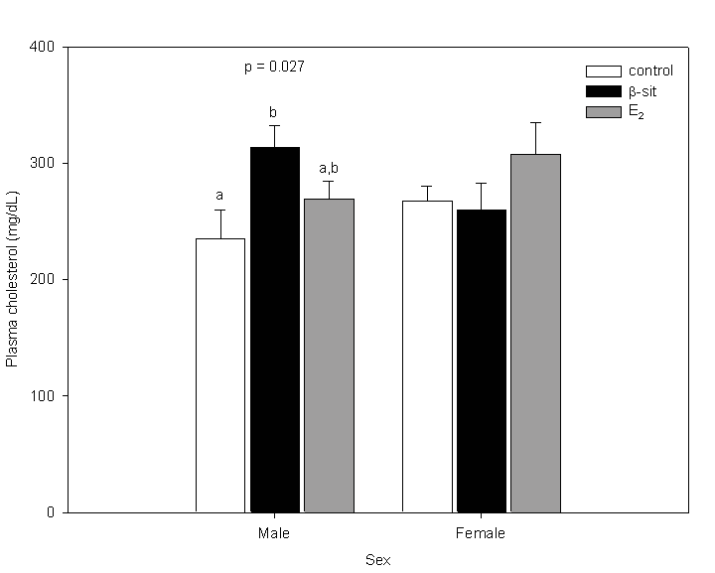
Plasma cholesterol concentrations (mg/dL) in male and female goldfish exposed to control, β-sit (200 μg/g) and E_2 _(10 μg/g) via Silastic implants for 21 days. Bars represent means ± SE. Different letters indicate treatments that are significantly different (p < 0.05).

Male β-sit-treated fish had significantly higher total plasma cholesterol concentrations than control fish (p = 0.027; Figure 5). There were no differences in female plasma cholesterol concentrations.

## Discussion

The *de novo *cholesterol biosynthetic pathway begins with the acquisition of acetyl-CoA, but commitment to cholesterol synthesis occurs subsequent to acetyl-CoA production. TG biosynthesis also utilizes acetyl-CoA as a substrate, potentially diverting substrate from the *de novo *cholesterol synthetic pathway [27]. TG is the primary metabolic energy storage molecule in fish where upwards of 80% of total body lipid composition is present as TG [7]. The current study suggests acetyl-CoA is preferentially directed towards TG synthesis over cholesterol in early to mid-recrudescing gonads of male and female goldfish. Ovarian tissue preferentially uses lipoprotein-derived cholesterol as a steroidogenic substrate [6], therefore, a large *de novo *cholesterol biosynthetic capacity was not expected in female gonads. The higher ^14^C-TG enrichment in ovarian tissue is consistent with an increased TG demand during reproductive development; growing oocytes incorporate high concentrations of TG to provide metabolic fuel for developing embryos [28]. Similar studies on trout (*Salmo gairdneri*) have shown high acetate incorporation towards TG synthesis during later gonadal development [45]. In testicular tissue it is believed that *de novo*-derived cholesterol is the primary substrate for steroidogenesis [2], therefore, more substantial ^14^C-acetate incorporation towards cholesterol was predicted. In contrast, ^14^C was enriched 6-fold and 3-fold higher towards TG and FFA than towards cholesterol and CEs. This higher level of *de novo *TG synthesis was consistent in all male treatment groups.

While fish testes generally have low lipid content that varies with season and reproductive stage [7], the high plasma T concentrations in male control fish suggest T synthesis (and thus steroidogenic substrate) was not impaired at the reproductive stage in the present study. The ^14^C-acetate incorporation data suggest testis tissue may direct acetyl-coA towards TG formation when cholesterol availability to steroidogenesis is not limiting. Plasma cholesterol concentrations were above 200 mg/dL for both sexes in all treatment groups, suggesting cholesterol and steroidogenic capacity were not limited at the point of circulatory uptake or *de novo *synthesis of cholesterol. The absence of a reduction in plasma T in the E_2 _treated fish and its significant decrease in β-sit exposed animals provides evidence of a unique, non-estrogenic mechanism of β-sit endocrine effects. Additionally, the depression of FFA synthesis by both β-sit and E_2 _treatment demonstrates common effects on lipid dynamics in exposed fish. Previous studies have established that β-sit changes plasma cholesterol dynamics and has endocrine effects distinct from E_2 _[29]. The regulation of steroidogenesis is a multifaceted feedback system among the hypothalamus, pituitary and the gonads, known as the HPG axis, therefore impairment of function can occur at multiple levels. MacLatchy et al. [28] demonstrated that β-sit does not alter plasma luteinising hormone (LH) concentration, while E_2 _interacts with the HPG axis extensively [30-32]. In particular, E_2 _and FSH are involved in regulating lipid accumulation in the ovaries of salmon (*Oncorhynchus kisutch*), thereby demonstrating a potential for E_2 _lipidemic effects [33]. The increase in plasma cholesterol seen currently in the male β-sit treatment group and an absence in the E_2 _treatment group, demonstrates independent effects of β-sit on plasma cholesterol concentration, likely caused by a mechanism distinct from an E_2 _feedback system. Evidence of β-sit interacting with lipid synthetic enzymes in humans [18] would suggest disruption likely occurs at the level of cholesterol biosynthesis. Further studies examining lipid synthetic enzyme kinetics will provide good insight towards understanding the interaction of β-sit and E_2 _with the cholesterol synthetic pathway.

The goldfish used in the current study were undergoing early to mid-recrudescence, based on preceding acclimation conditions, observations during sampling, GSI values and plasma T concentrations. Reproductive development in goldfish is highly regulated by changes in environmental parameters such as photoperiod and water temperature [34] and the laboratory environmental conditions for this study were designed to initiate recrudescence (15–16°C; 14 h L:10 h D). Fish experience low circulating cholesterol concentrations during sexual maturation, and it is known plasma lipids are variable and influenced by reproductive state [10]. Testis lipid content varies with season and, therefore, reproductive development [7]. It is thus likely that sensitivity to lipid-altering chemicals may also vary with reproductive state, presenting contradicting results dependent on the specific physiology and lipid metabolism of the particular gonadal stage at the time of exposure. This appears to be the case with β-sit, which affects endocrine endpoints such as plasma T and cholesterol concentration differently in males and females at different reproductive states and exposure durations. For example, in a long-term β-sit exposure covering a 5-month period of gonadal development and starting prior to the initiation of recrudescence, there were no effects on plasma T or cholesterol concentration by β-sit during recrudescence [35]. However, at the initiation of a subsequent cycle of gonadal development, in the same 5-month exposure, male fish experienced significant reductions in plasma T [36]. In the current study, plasma cholesterol concentrations increased in the β-sit treatment group, an effect in contrast to previous reports [14,15], indicating β-sit can also have variable effects on plasma lipids. It is, therefore, critical to interpret endocrine effects of β-sit exposure with conscience to the reproductive stage at the time of exposure and sampling. Further, the complex and intricate nature of the regulation of steroidogenesis by the HPG axis makes the interpretation of plasma hormone data challenging. The reduction in plasma T in male fish exposed to β-sit but not E_2 _likely results from different sensitivities of the HPG axis to estrogenic control at different stages of the reproductive cycle; male and female fish were not sensitive to down-regulation of T synthesis by E_2 _in the present study. The impact of β-sit exposure on plasma T would, therefore, appear to be caused by non-estrogenic mechanisms, likely related to limited cholesterol availability to the steroidogenic pathway [16]. Steroidogenic acute regulatory protein (StAR) has been identified as a mitochondrial cholesterol transporter [37], and β-sit has been shown to reduce StAR mRNA abundance in male goldfish [36]. Given that *de novo *cholesterol synthesis was unaffected by β-sit exposure and plasma cholesterol concentrations were not decreased, it is highly possible that cholesterol delivery to the steroidogenic pathway is impaired rather than intracellular cholesterol availability.

Male and female goldfish responded differently to β-sit and E_2 _exposure; plasma testosterone and cholesterol concentrations were unchanged by β-sit in female fish. Few studies have examined the responses of both male and female fish to E_2 _or β-sit exposures. Similar steroid responses in male and female goldfish (male GSI = 3.9 – 4.9%, female GSI = 4.5 – 6.2%) to varying concentrations of sitosterol have been reported [29]; both sexes experience significant reductions in circulating T in response to β-sit, but not E_2_. In contrast, the present research found male fish to be more sensitive to reductions in plasma T following β-sit exposure than females. These differences in plasma hormone response between studies is likely at least partly due to differences in gonadal stage at the time of the exposures (current experiments, male GSI = 0.7–1.78%, female GSI = 1.9 – 2.8%) and, therefore, a different physiological environment in which exogenous chemicals interact with gonadal tissue. Further, circulating E_2 _is a normal physiological reproductive stage-dependent phenomenon in female fish [38], therefore feedback systems and metabolic responses to exogenous E_2 _exist in females that are not normally activated or required in male fish. In the present study, however, male fish were more sensitive to β-sit than female fish, and generally neither plasma T or cholesterol were sensitive to E_2 _in either sex. It appears there are sex differences in sensitivity to β-sit with regard to plasma hormones and cholesterol and these varying sensitivities are likely related to reproductive stage and the associated endogenous regulation of gonadal development.

A quantitative, physiologically-relevant indication of steroidogenic output can be determined using gonadal *in vitro *incubation methods that measure steroidogenic output by gonadal tissue [39]. Reported rates of T production in goldfish testis range from 1 pg/g (unknown GSI) [40] to 20 pg/mg (GSI 2.2%), with the latter corresponding to a plasma T concentration of 3.0 ng/mL [14]. *In vitro *steroid biosynthetic capacity was not measured in the current study due to limited gonadal tissue availability; however, control male plasma T concentrations were comparable to those reported by MacLatchy & Van Der Kraak [28]. Therefore, an estimate of the cholesterol requirements for T production in the male control group of the present study can be calculated using the *in vitro *biosynthetic capacity reported by MacLatchy and Van Der Kraak (20 pg/mg over an 18-h period). Cholesterol is used for additional cellular functions in the testis (storage, plasma membrane integrity, synthesis of other steroid hormones), therefore, any estimation of cholesterol requirements based solely on T production will be highly underestimated. Given this limitation, an estimate of 2.9 × 10^-10 ^moles of cholesterol were required to produce 20 pg of T (using a 1:1 testosterone: cholesterol stoichiometry). In the current study, control male fish incorporated 1.47 × 10^-9 ^moles of acetate, or 8.8 × 10^14 ^molecules towards cholesterol synthesis during the 18-h incubation period. Given an 18:1 stoichiometry (18 molecules of acetate for each cholesterol synthesized) [41], this equates to 8.2 × 10^-11 ^moles of cholesterol synthesized *de novo*. Additional studies have measured gonadal tissue cholesterol concentrations from recrudescing male goldfish and report 22.6 mg cholesterol/g tissue [35]. This equates to 5.8 × 10^-5 ^mol/g, which if corrected to 25 mg of testis tissue as used in the current study, estimates 1.5 × 10^-6 ^mol of endogenous cholesterol is present in each *de novo *testis sample. Therefore, the *de novo *contribution (8.2 × 10^-11^) is very small relative to the total cholesterol pool (1.5 × 10^-6^). However, the estimated *de novo *synthetic capacity is within an order of magnitude of the estimated steroidogenic needs for T synthesis (2.9 × 10^-10^) and could make a significant contribution to the steroidogenic substrate pool.

Identification of lipid classes was limited to cholesterol, TG, FFA and CE; it is highly likely that ^14^C-acetate was incorporated to phospholipids and monoacyl- and diacyl-glycerols as well [26]. Weigand and Idler [45] examined acetate incorporation to various lipid classes in ovarian tissue of trout and found that while polar lipids were favoured when GSI was small, TG was preferentially synthesized in fish with larger GSI. In catfish (*Heteropneustes fossilis*), plasma and ovarian phospholipid concentrations followed the same trends as cholesterol and TG throughout the reproductive cycle [42] and high plasma phospholipid concentrations have been documented during maturation in goldfish [43]. Given such indications that dominant gonadal lipid classes can vary throughout the reproductive cycle, it should be noted that broad conclusions on gonadal lipid synthesis are limited to only the neutral lipid classes in the current study.

The high ^14^C-acetate incorporation towards FFA in control fish was absent in the corresponding male β-sit and E_2 _treatment groups. Phytosterols detrimentally affect cholesterol metabolism in the human disease sitosterolemia by inhibiting endogenous cholesterol biosynthesis [19], therefore, inhibitory impacts of sitosterol exposure on fish were expected. The first enzyme subsequent to the formation of acetyl Co-A in the cholesterol biosynthetic pathway, acetoacetyl CoA thiolase, is down-regulated in sitosterolemic patients [18] and phytosterols have been shown to down-regulate numerous other cholesterol biosynthetic enzymes in mammals [44]. The current study did not examine enzyme kinetics. The three week β-sit exposure prior to gonadal tissue incubation with ^14^C-acetate would serve to establish physiological conditions similar to those in sitosterolemia, where tissues were exposed to higher than normal concentrations of plant sterols. Surprisingly, there were no differences in ^14^C-enrichment of cholesterol or CEs in male or female fish, indicating no changes in *de novo *cholesterol synthetic capacity among treatment groups. The lower ^14^C-acetate incorporation to FFA in male β-sit- and E_2_-treated gonads suggests both substances have the capacity to down-regulate endogenous FFA synthesis in gonadal tissue. Further, the similar effect by both substances demonstrates the capacity of β-sit to act with estrogenic properties *in vivo*. Therefore, while the current study provides evidence that β-sit has non-estrogenic endocrine disrupting behaviour, as evidenced by decreased plasma T, it also has effects similar to estrogen in terms of its effects on FFA synthesis in the gonads. There is no evidence in the present study to suggest β-sit has effects on *de novo *cholesterol biosynthesis at this reproductive stage for either sex; there is no variation in cholesterol synthesis in the gonads of fish exposed to β-sit or E_2_.

## Conclusion

The present study clearly demonstrates that ovarian and testicular tissues have comparable capacities for *de novo *cholesterol synthesis at early to mid-recrudescence, and neither β-sit nor E_2 _treatment disrupts gonadal cholesterol biosynthesis in goldfish at this reproductive stage. Further, differences between β-sit and E_2 _exposure effects were demonstrated, suggesting the mechanisms of action of β-sit occur at points outside the HPG axis to yield effects on steroid biosynthesis and lipid metabolism.

## Authors' contributions

RLS originated the detailed experimental design, some of the study concepts and methodology; carried out the experimental exposures, steroid and lipid analyses, statistical analyses; and drafted the manuscript. MD participated in the infrared spectroscopy validation of lipid types. DLM initiated the original study concepts, participated in design of the study and helped to draft the manuscript. All authors read and approved the final manuscript.
